# A mixed-methods protocol for identifying successful sustainability strategies for nutrition and physical activity interventions in childcare

**DOI:** 10.1186/s43058-021-00108-x

**Published:** 2021-01-14

**Authors:** Taren Swindle, Dong Zhang, Susan L. Johnson, Leanne Whiteside-Mansell, Geoff M. Curran, Janna Martin, James P. Selig, Laura L. Bellows

**Affiliations:** 1 Department of Family and Preventive Medicine, University of Arkansas for Medical Sciences, 4301 W. Markham St, #530, Little Rock, AR 72205-7199 USA; 2 Department of Family and Preventive Medicine, University of Arkansas for Medical Sciences, 4301 W. Markham St, #530, Little Rock, AR 72205-7199 USA; 3 Department of Pediatrics, University of Colorado Anschutz Medical Campus, 12700 East 19th Avenue Box C225, Aurora, CO 80045 USA; 4 Department of Family and Preventive Medicine, University of Arkansas for Medical Sciences, 4301 W. Markham St, #530, Little Rock, AR 72205-7199 USA; 5 Department of Pharmacy Practice and Psychiatry, University of Arkansas for Medical Sciences, 4301 W. Markham St, #522-4, Little Rock, AR 72205-7199 USA; 6 Department of Family and Preventive Medicine, University of Arkansas for Medical Sciences, 4301 W. Markham St, #530, Little Rock, AR 72205-7199 USA; 7 Department of Biostatistics, University of Arkansas for Medical Sciences, 220 UAMS Campus Dr., #781, Little Rock, AR 72205-7199 USA; 8 Division of Nutritional Sciences, Cornell University, Martha Van Rensselaer Hall, Ithaca, NY 14853 USA

**Keywords:** Sustainment, Nutrition, Physical activity, Implementation science, Childcare

## Abstract

**Background:**

Despite the importance of sustainability for nutrition and physical activity in public health interventions, limited studies have explored the factors that promote and inhibit evidence-based program sustainment in the childcare setting. This study protocol describes a mixed-methods approach to develop novel sustainability strategies based on real-world settings and stakeholder feedback, with the goal of providing support for future obesity prevention programs and related studies on intervention sustainability. Two interventions, Together, We Inspire Smart Eating (WISE) and The Food Friends’ (FF) Fun with New Foods and Get Movin’ with Might Moves, are studied to this end.

**Methods:**

The study will deploy an explanatory, sequential mixed-methods design. First, the research team will collect a quantitative survey to assess rates of sustainment among WISE and Food Friends sites. We expect to collect 150 surveys from WISE and FF sites combined. Data from these surveys will be used to purposively sample sites for 12 to 18 site visits. Specifically, we will purposively sample low, partial, and high sustaining sites where we will conduct key informant interviews and focus groups as well as validate self-reports on sustainability. Survey content, qualitative interviews, and coding will be based on the Dynamic Sustainability Framework. We will draw on findings from the quantitative survey on predictors of sustainment and the qualitative site visits to understand varying levels of program sustainment. Then, we will utilize evidence-based quality improvement sessions to engage stakeholders in developing a multi-component sustainability strategy.

**Discussion:**

This study will provide a stakeholder-informed sustainability strategy ready for testing in a full-scale trial examining effects on sustainment of evidence-based nutrition and physical activity practices in childcare. We expect this strategy to be relevant for educators and consistent with the views of administrators as a guide for future practice for the targeted nutrition and physical activity interventions and beyond.

Contributions to the literature

- Identify factors that promote and inhibit nutrition and physical activity program sustainment in childcare.
- Explore programs at distinct levels of sustainment and inform design of strategies for sustaining evidence-based nutrition and physical activity interventions in childcare.
- Provide a foundation for designing and testing sustainment strategies in future studies—applicable to WISE and FF and a range of other potential interventions.

## Background

Children between 2 and 5 years of age in the USA rarely meet dietary recommendations, and most fall short of achieving recommended levels of physical activity [1–4]. Further, 40% of children are overweight or obese by age 5 [5]. Such excess weight increases the risk for metabolic syndrome, cardiovascular disease, obesity, and some cancers in adulthood [6, 7]. Consuming a healthy diet (e.g., fiber and antioxidant-rich) [8–10], engaging in physical activity (PA), [10–12], and maintaining a healthy weight [10, 13, 14] provide significant protection against disease. Given that early life patterns track into adulthood, [15, 16] early childhood is an important time period for such prevention efforts. Because a large majority of young children in the USA spend an average of 30 h per week or more in early care and education programs [17], childcare is a promising setting for implementing and sustaining nutrition and physical activity programs.

### Importance of sustainment for disease prevention

Sustainability is both (a) the process by which an innovation becomes integrated into existing systems [18] and (b) an outcome in which an innovation is sustained after initial implementation or funding ends [19]. Sustainability is a key target outcome for public health interventions and programs to have the desired effects on population health [20]. Yet, the sustainability of public health programs varies, with estimates indicating that 40 to 60% of programs cease or operate at lower levels after initial funding is withdrawn [21–25]. Thus, sustaining evidence-based programs in the community setting is a significant challenge, undermining investments in implementation. Reflective of this gap, leaders in the field of implementation science have identified sustainability as a priority area for future studies [26].

### Target interventions to inform sustainment in childcare

Sustaining effective programs for nutrition and physical activity in childcare would promote disease prevention in a setting serving a large number of children; yet, little is known about factors that predict sustainability (or lack thereof) [27, 28]. Two existing interventions in the childcare setting provide an opportunity to study factors associated with sustainment in childcare—Together, We Inspire Smart Eating (WISE)® and Food Friends® (FF; Fun with New Foods and Get Movin’ with Mighty Moves). Both interventions include three elements: (a) a classroom curriculum, (b) educator training, and (c) parent education using materials for outreach [29].

WISE was designed to establish healthy early eating habits with food experiences and supporting activities executed weekly for children 3 to 8 years old across a 9-month school term in Arkansas. WISE has been disseminated for approximately 7 years. WISE has created positive changes in child and family eating behaviors that align with the current recommended Dietary Guidelines for Americans, specifically, increasing reported consumption of WISE fruits and vegetables, more fruits and vegetables in general, and decreasing intake of nutrient-poor food compared to children not experiencing WISE and before exposure to WISE [30, 31].

FF is a preschool program designed to address childhood obesity by establishing healthful eating and physical activity behaviors in preschool-aged children. The program consists of two components (1) Fun With New Foods®, a 12-week intervention, which focuses on helping children increase their willingness to try new foods, and (2) Get Movin’ With Mighty Moves®, an 18-week intervention, which aims to enhance preschoolers’ gross motor skill development. The program is based on constructs of the social cognitive theory, tenets of social marketing, and is embedded within Bronfenbrenner’s social ecological framework [32]. FF was implemented mainly in Colorado for over 20 years and has successfully demonstrated increases in both children’s willingness to try new foods (food preference) and gross motor performance in preschool-aged children in the short term [33, 34] and longitudinally [35, 36].

The current study aims to understand sustainment factors common across and unique to each intervention. We expect examining similarities and differences across two programs will contribute to knowledge that will be more generalizable to promote sustainment for a range of evidence-based practices and interventions in childcare. To this end, we will complete two aims:

#### Aim 1: Identify factors associated with sustaining nutrition/PA programs in childcare settings

We will use an explanatory, sequential mixed-methods design to (a) collect survey data and conduct multivariate analyses to understand predictors of sustainability and (b) conduct site visits to perform key informant interviews and focus groups; validate self-reports on sustainability; identify environmental contributors to sustainability; and document program adaptations. We will also conduct interviews with external stakeholders to understand decisions affecting support for nutrition/PA programs.

#### Aim 2: Develop a multi-faceted sustainability strategy with stakeholder input

We will use evidence-based quality improvement (EBQI) sessions [37–39] to engage stakeholders in developing a multi-component sustainability strategy. Data from aim 1 on the predictors of sustainability and innovation from sites with high sustainment will be used to inform stakeholder-selected strategies.

### Theoretical framework

The proposed project will use the Dynamic Sustainability Framework (DSF) [40] to understand predictors of sustainability for interventions in childcare and to design sustainability strategies to be tested in this setting. The DSF posits that characteristics of the intervention (e.g., components and practitioners), the context (e.g., climate and staffing), the larger ecological system (e.g., policy and population characteristics), and the fit among these elements contribute to sustainability outcomes. Consistent with our focus on adaptation, the DSF recognizes that adaptation is inevitable and may be key to enhancing long-term sustainability. In the proposed study, this framework will inform the selection, adaption, and development of quantitative and qualitative measures in aim 1. Additionally, we will apply the Wiltsey-Stirman framework [41] for coding program modifications and adaptations to qualitative data gathered in aim 1. The Expert Recommendations for Implementing Change (ERIC) taxonomy of implementation strategies [42] will be used as a starting point for developing and selecting sustainment strategies in aim 2. The integration of these will provide a solid foundation for the proposed research.

## Methods

### Aim 1

#### Study design

**Fig. 1:**
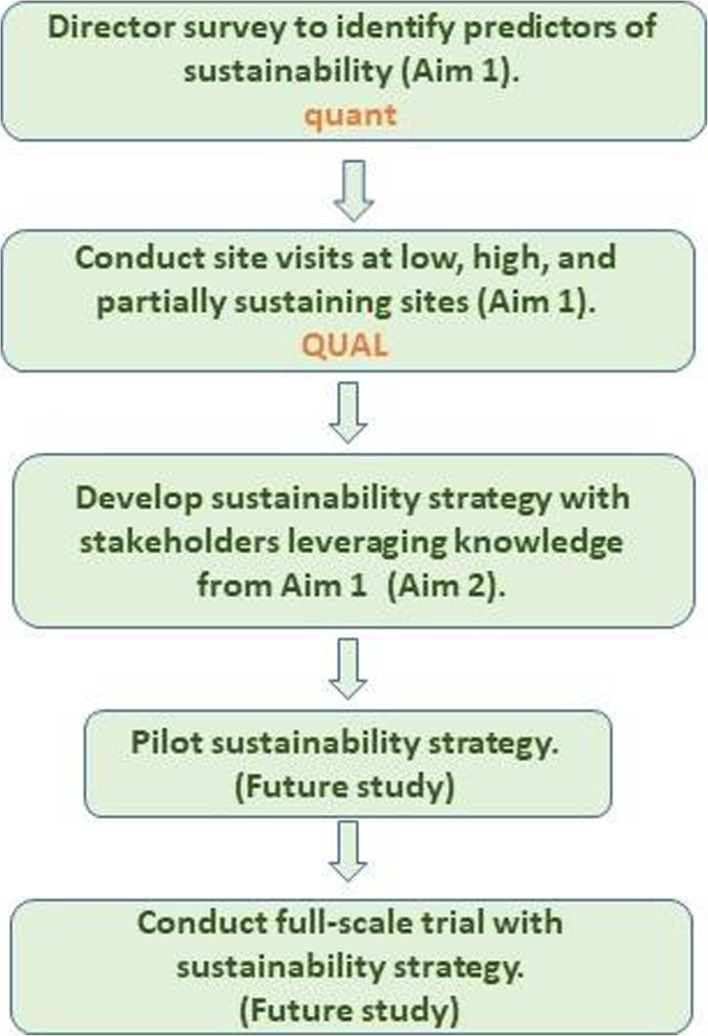
Study activities and future plans

Figure 1 provides an overview of study activities. All study procedures will be conducted in accordance with protocols approved by the University of Arkansas for Medical Sciences Institutional Review Board (IRB). Aim 1 of the current study will identify predictors of sustainability unique to each program and common across programs. We will focus on three aspects of sustainability: (1) “continued attention” to nutrition and physical activity promotion and obesity prevention [20], (2) the degree to which centers use original program components [20, 43], and (3) the extent and nature of modifications to components [43]. We will also examine different levels of sustainability: high, partial, and low (defined below). Our multi-pronged approach recognizes that sustainment is not a dichotomous outcome, and partial sustainment [43–45] is likely common. Thus, we will focus both on which aspects of each program are sustained in the current school year and to what degree they are sustained (e.g., frequency, length, and fidelity). First, we will use a quantitative survey to identify predictors of self-reported sustainability. Then, we will conduct purposive site visits to (1) validate the degree of sustainability with in-person fidelity assessments, (2) collect in-depth information to understand local-level decision making relative to sustainability, (3) identify and categorize adaptations to the programs, and (4) document strategies used by high sustaining programs which could be tested in the future.

We will use an explanatory, sequential mixed-methods design to understand barriers and facilitators specific to sustainability (quant→QUAL) [46, 47]. See Table 1 for data sources for aim 1. First, we will complete a director survey of current sustainability. Quantitative data will be gathered with an online REDCap survey of directors from FF and WISE sites (*N* = 373 potential sites; *N*_FF_ = 210; *N*_WISE_ = 163). Because directors approve lesson plans and curricular purchases, they can report on program activities. We have set the parameters of the sampling frame for the director survey to be receipt of Child and Adult Care Food Program support (a proxy for serving families affected by low income) and being trained (or retrained) between 2010 and 2017. Notably, our databases include data on variables such as dates of training. The director survey will incorporate scales from the established Program Sustainability Assessment Tool (PSAT) [48] to measure funding/resource stability, community partnerships, organizational capacity, program evaluation and adaptation, communications, and strategic planning. The PSAT was developed for public health programs, but we will adapt it via cognitive interviews (*n* = 5) with teachers to be retrospective and to reflect the childcare setting. The survey also will use established measures to assess the organizational climate [49], perceptions of the innovation [50], and reported fidelity to core components [51].

**Table 1:** Data sources for aim 1

Source	Information	Dynamic Sustainability Framework constructs
Director surveys	PSAT (adapted); current nutrition and PA programming	Intervention (components); context (training)
Site visits	EPAO (subset of modules); adaptation categorization; program fidelity	Intervention (delivery); context (culture)
Director interviews	Sustainability decision making (local); institutional knowledge; barriers and facilitators to sustainment	Context (information systems); ecological system (policy and regulations)
Mini focus groups	Values; organizational structures; barriers and facilitators to sustainment	Intervention (practitioners); context (culture)
External stakeholder interviews	Factors influencing funding and policy priorities/shifts; decisions to cut/cease program support	Context (business model); ecological system (market forces and policy)

*PSAT* Program Sustainability Assessment Tool, *EPAO* Environmental and Policy Assessment and Observation

The survey will begin with questions about current nutrition/PA programming using broad questions and then, via branching logic, specific questions (program component usage; investment to sustain programming; use of technical assistance, follow-up training, contact with program administrators; and plans for future use). From this information, we will determine three categories of program sustainability: (1) *high* sustainability programs continue to implement core components with or without appropriate adaptations; (2) *partial* sustainability programs use some, but not all, components; and (3) *low* sustainability programs no longer use FF/WISE or exhibit continued attention to nutrition/PA programming.

Directors will be invited by email to complete the survey online. Completers will be entered into 1 of 15 drawings for $100. We expect 150 responses (40%) based on our experience in recruiting childcare participants. We will use additional strategies to increase participation (e.g., phone calls, mailed flyers, visits) if we cannot reach our target response rate [47]. For example, we will draw on relationships with county-based Extension educators, early childhood councils, and Child Care Resource and Referral agencies. We will also make presentations at state conferences and meetings to engage with potential participants.

Qualitative data will be collected based on survey data. Sites will be purposively sampled (criterion-i) [52] to reflect the 3 categories of sustainability. We expect to target 2–3 sites per category in Arkansas (AR) and Colorado (CO). Thus, our total sample will include 12–18 sites split evenly across AR and CO and across sustainment categories. During site visits (2–3 days), the research team will conduct semi-structured interviews with directors and mini focus groups with educators (groups of 4–6 per Krueger and Casey) [53, 54]. Director interviews will include questions about external factors influencing sustainability decisions (e.g., regulations, funding). We will query whether institutional knowledge about FF/WISE was transferred during leadership changes and how decisions to sustain (or not) were made. Mini focus groups with teachers are cost-effective and allow them to share experiences without directors present [54]. Interviews and focus groups, informed by the DSF, will ask about contextual elements (e.g., values, organizational culture) that influenced sustainability decisions, processes in place to support sustainability, and the decision-making process to reduce, abandon, or adapt use of the program.

We will also engage relevant external stakeholders in each state for in-depth interviews to understand external factors affecting sustainability. Targets include delegates from the state Departments of Education and Health, leaders from prior funding agencies, members of policy councils, and agency administration (which provides leadership beyond site-level directors). We expect to interview 3 to 5 external stakeholders per state. Interview guides for mini focus groups and interviews will align with constructs of the DSF [40] and be modified as needed [54]. To ensure the validity of the data, the research team will follow a standard training protocol, including multiple rounds of mock interviews [55]. Interviews and mini focus groups will last 30 to 60 min and be transcribed verbatim. Interview and focus group participants will receive a $25 incentive.

Observational data will include observer field notes [54, 56] and two quantitative measures, the Environmental and Policy Assessment and Observation (EPAO) [57] and an assessment of fidelity tailored to each program [58]. The EPAO, a reliable and valid measure of childcare nutrition and PA environments, will provide a standardized assessment of contextual factors that could support or interfere with the sustainability of the FF and WISE programs. We will prioritize EPAO modules that measure programmatic practices (e.g., planned nutrition and PA lessons) and behaviors that best capture influence of the program or training (e.g., provider interactions). We will exclude less relevant details (e.g., condiment use). The PIs will train research staff using the published online EPAO modules and certification tools [59]; research staff will also be trained to document observable characteristics that are important to program implementation (e.g., program materials present and interpersonal climate). Notable adaptations of the program will be analyzed with the Wiltsey-Stirman framework [41]. Researchers will also document the use of skills (taught by the programs) outside of programmatic lessons (e.g., encouraging sensory exploration of foods). Finally, the fidelity measure will assess the quality and dosage of program components during a classroom lesson (e.g., engagement with mascots, frequency of role-modeling) and build on our own published measures [58].

#### Participants, setting, and sample size

All directors and educators previously trained in FF or WISE will compose the potential participant pool for aim 1 surveys, interviews, and focus groups. We will recruit participants through contacts (first) from research staff and from county Extension or childcare resource and referral agents (second) to ensure representation across racial/ethnic groups and geographic regions of AR and CO. Purposively sampled sites for site visits will be recruited into the study through informational meetings and IRB-approved consenting procedures. Participation in aim 1 activities will be short term and will not require continued engagement after the initial data collection.

#### Aim 1 data analysis

Surveys will provide quantitative data on (1) the levels of sustainment across programs and components, (2) factors unique and common to predicting sustainability, and (3) the moderating effect of length of sustainment (lag) on identifying salient predictors of sustainability. We will examine the data for missing values, extreme scores, and the shape of distributions for each variable. Outliers will be excluded and will use full information maximum likelihood in analyses to manage missing data greater than 5%. We will also compare item and summary scores across program types to identify systematic differences between nutrition programs. The primary analysis will use a general linear model (multiple regression) with sustainment scores as the outcome and the following predictors: PSAT scores, program type, context characteristics (internal, external, and program factors), time since program implementation (lag), and the interactions of PSAT scores and lag. A statistically significant (*p* < .05) result from the *t* test for each predictor will be used to identify factors associated with program sustainability. We will also determine if there are interactions between PSAT scores and program type that suggest different models of sustainment for FF vs. WISE (*p* < .05). Assuming a response of 40% from our participant pool, 150 sites will be represented in the director survey. With *N* = 150 (two-sided *α* = .05), we would have 80% power to detect a Pearson correlation of 0.23. For comparisons of program type, assuming *n*_WISE_ = 45 and *n*_FF_ = 105 (two-sided *α* = .05), we would have 80% power to detect a Cohen’s *d* of 0.5 or a medium-sized effect. In the proposed multiple regression (two-sided *α* = .05, 10 predictors), we would have 80% power to detect an *f*^2^ effect size of 0.05 (a little larger than a “small” effect) [60]. For context, with a base model accounting for 25% of response variance, we would have 80% power for an added predictor accounting for an additional 3.9% of variance. These power estimates apply for tests of both main effects and interactions. Recruiting 30% of our participant pool (*N* = 112) would provide 80% power to detect a Pearson correlation of 0.26, an *f*^2^ effect size of 0.07, and a Cohen’s d of 0.54 for group comparisons, assuming *n*_WISE_ = 49 and *n*_FF_ = 63.

Qualitative interviews with targeted sub-groups will provide information on the barriers and facilitators to sustainability specific to each program and for those that co-exist across programs. Further, we will be able to compare self-reported perceptions of sustainability with observational data and to collect data on how agencies decide to reduce, abandon, or adapt the use of a program. Transcripts will be coded by the PIs and trained research assistants using directed content analysis [61]. Observer field notes will be matched to interviews. Nvivo software will be used for team coding. The DSF [40] will provide sensitizing concepts to build initial codes that will succinctly label significant, recurrent ideas across participants. Analysis will focus on identifying common and distinct themes across the three categories of sustainability. Inductive codes will be incorporated into the codebook as they develop [62]. Primary and secondary coders (at least one each from AR and CO) will code the same transcripts until inter-rater reliability is established. Afterward, coding will be independent, but we will hold weekly consensus meetings to reduce bias and ensure reliability and validity. At weekly meetings, disagreements will be resolved, and the codebook will be expanded as needed. Summaries of site-level findings will be presented back to participants for member checking.

### Aim 2

#### Study design

We will use a stakeholder-driven process called evidence-based quality improvement (EBQI) [37–39, 63–66] to ensure that our sustainability strategy reflects cultural needs and preferences. EBQI has been used frequently to develop strategies and adapt interventions in primary care, outpatient care, and pharmacy practice [38, 64, 67, 68]. Consistent with the DSF [40], we expect that engaging stakeholders will contribute to the most effective strategies. EBQI panels will operate according to principles of community-based participatory research [69] and best-practices for engaging stakeholders in implementation [70]. Childcare directors and educators will contribute the local knowledge needed to select and tailor strategies; the research team will provide expertise on implementation approaches.

The EBQI process will (1) leverage strategies that worked for high sustainability sites from aim 1, (2) prioritize barriers and facilitators to sustainability reported from sites and external stakeholders from aim 1, (3) identify theoretically informed sustainability strategies that match priorities/needs, and (4) tailor sustainability strategies to the early childhood context. The EBQI panel will include 8 to 12 stakeholders combining FF and WISE users, and the diversity of the panel will reflect the diversity of the target populations.

EBQI is a flexible process conducted across a series of meetings with topic-driven agendas; each session will last 2 h. *In EBQI session 1*, the research team will present a summary of aim 1 findings, conduct a “member checking” exercise to assess validity of the findings, reach consensus on prioritized barriers and facilitators to inform the sustainment strategies, and vet sustainment strategies identified in aim 1. *In session 2*, we will present potential sustainment strategies mapped by the research team to the ERIC [42] taxonomy of implementation strategies, incorporating knowledge on strategies that worked in high sustainability sites and predictors of sustainability (from aim 1). To reach a consensus on sustainment strategies, we will use techniques outlined by Powell et al. [71], including concept mapping with virtual polling. This method provides quantifiable information and promotes the efficient collection of input in real time. Our existing relationships with Extension offices and regional medical facilities will facilitate virtual meetings in all counties. *In session 3*, we will present the draft strategies/tools, collect feedback for revisions, and receive final approval. *In sessions 4 and 5*, we will present our plan for a future pilot test of the sustainability strategy to assess acceptability and adoption. Stakeholders will receive $50 per session. By applying the EBQI process to the study of sustainability rather than implementation, our findings may be broadly applicable to the field of implementation science.

#### Participants, setting, and sample size

We will engage key community stakeholders to serve on our EBQI panel and provide input on the development of a sustainment strategy. This process will review the existing scientific evidence and data from aim 1 with the EBQI panel to solicit stakeholder input on how best to sustain nutrition/PA programs in childcare. EBQI sessions will be audio-recorded so that we can review the content covered in each session and rapidly code the reactions of the EBQI panel. These data will be collected anonymously. Panel members may also be asked to complete survey instruments to provide quantitative input on proposed strategies. The identity of EBQI members will not be included in reports or manuscripts. We expect to engage up to 12 stakeholders for the panel. The EBQI panel will meet 5 times virtually.

For aim 2, stakeholders and pilot participants will be recruited through IRB approved procedures, focusing on agencies with which we have continued partnerships. We expect these activities to include site visits, emails, contacts at early childhood conferences, and phone calls. Stakeholder recruitment will reflect the demographics of early-childhood educators in each state as published by the state departments of education. External stakeholders will be recruited through established relationships with governing, funding, and administrative bodies in each respective state.

#### Data analysis

We will use an online platform and database server to collect and store the EBQI panel’s perceptions of feasibility and importance of potential sustainment strategies. Then, we can query the database [72] in real time to plot potential strategies by their rated importance (*x*-axis) and feasibility (*y*-axis), consistent with a concept mapping approach [71]. Strategies above the mean for both criteria will be considered for inclusion in the sustainability strategy. After each EBQI session, research team members will document what they observed and heard, what was resolved, and what remains undecided. These meeting minutes will guide subsequent EBQI sessions. The research team will also assimilate panel feedback, translate it to actionable plans, and develop the next iteration of materials for panel input. Qualitative information from meeting minutes and audio recordings will be analyzed using directed content analysis [61] relative to the main goals of the EBQI process (e.g., matching barriers/facilitators to sustainability strategies). After data from the final EQBI meeting are analyzed and incorporated into the design, the sustainability approach will be fully specified.

## Discussion

This study will help address critical gaps in knowledge about the extent to which nutrition education and PA programs are sustained in childcare settings and the predictors of sustainability (internal and external factors within/between intervention programs). The results of this study are likely to provide useful strategies for sustaining evidence-based programs in childcare, as well as in other settings (e.g., home visiting, worksites, and schools). Successful implementation of sustainability strategies will inform other projects as to the process of engaging stakeholders to inform the selection and development of optimal sustainability strategies, an essential approach for sustaining program impact [73, 74]. Finally, our study will provide a “follow up of factors that contribute to the sustainability of evidence-based interventions in public health” and an opportunity to study the relationships between the “context and local capacity of clinical and community settings” and sustainability [75]. This addresses a critical gap in public health to identify and prioritize strategies to promote sustainability [76].

Several factors make our study of sustainability unique. First, we will study two interventions simultaneously—each in different stages of its life cycle. Specifically, FF has ended dissemination efforts after 20 years. However, some sites continue to use the program despite workforce turnover and withdrawal of state-level support. Analyzing the life-cycle of FF will help us understand and contribute to the design of novel sustainability strategies and ensure the full benefit of investments in newer programs (such as WISE). Second, our study will be the longest-range view of sustainability to date (over 20 years for some Food Friends sites), providing a unique opportunity to study long-range predictors of sustainability. In addition, our study will provide an opportunity, through site visits, to assess unintended consequences of sustainment, and the ability to compare self-reported and observed measures of sustainability. Furthermore, the proposed project will provide data about successful adaptations that have been made to programs to promote sustainability and how these decisions were made.

## Challenges and limitations

To include a pilot study of the sustainability strategy would be ideal; however, this is beyond the scope of the current study. Alternatively, we will dedicate a follow-up study to a robust pilot of sustainability strategies before a future, large-scale implementation trial. An additional potential problem is coordinating the stakeholders across Arkansas and Colorado. However, the study team has experience managing the logistics of stakeholder panels with diverse members conducted via virtual meeting. Lastly, the COVID-19 pandemic has resulted in significant changes to the research and childcare landscape. For example, over 45 sites trained in WISE have closed permanently. We have included power estimates for a reduced sample size should COVID-19 affect our ability to recruit participants. Further, the research team will remain responsive to COVID-19 conditions in our methodologies (e.g., virtual site visits versus in-person site visits as proposed).

## Conclusion

We expect our study to produce a multi-faceted package of sustainability strategies based on real-world settings. Our stakeholder-informed sustainability strategy is anticipated to be relevant for educators and consistent with the views of administrators. With the combination of mixed-methods approach, stakeholder engagement, and analysis across multiple interventions, our study has the potential to contribute towards the advancement the science of sustainability.

## Data Availability

The datasets used and/or analyzed during the current study will be available from the corresponding author on reasonable request.

## Abbreviations

PA: Physical activity
FF: Food Friends, Fun with New Foods & Get Movin’ with Mighty Moves
WISE: Together, We Inspire Smart Eating
EBQI: Evidence-based quality improvement
DSF: Dynamic Sustainability Framework
ERIC: Expert Recommendations for Implementing Change
IS: Implementation science
PSAT: Program Sustainability Assessment Tool
EPAO: Environmental and Policy Assessment and Observation
